# Concussion management and concussion recovery in Gaelic games: a qualitative analysis

**DOI:** 10.3389/fspor.2024.1470358

**Published:** 2024-09-27

**Authors:** Ed Daly, Lisa Ryan

**Affiliations:** Department of Sport Exercise and Nutrition, Atlantic Technological University, Galway, Ireland

**Keywords:** recovery, concussion, Gaelic games, brain injury, education

## Abstract

**Background:**

The purpose of this qualitative research study was to interview current and retired Gaelic games athletes to understand the current landscape of concussion recovery in Gaelic sports from the athlete perspective.

**Methods:**

A grounded theory methodology was employed to explore the experiences of a cohort of Gaelic games athletes (*n* = 22) regarding recovery from concussion, the levels of concussion awareness in Gaelic sports and their opinions on current concussion identification protocols. The study's data were gathered through semi-structured interviews.

**Results:**

Two major themes were identified, (1) Male and female athletes experience a range of acute and chronic symptoms post-SRC and (2) Gaelic sports athletes are expected to demonstrate constant allegiance and commitment to the GAA. These themes were further divided into categories and subcategories.

**Conclusion:**

Based on the experiences of the cohort of Gaelic sports athletes, there exists a wide variation of SRC symptomology in the acute, and chronic (post-concussion syndrome) phases. In many cases, there are reports of long-term side effects associated with the perceived mismanagement or misdiagnosis of SRC in Gaelic sports. Gaelic sports athletes require a more robust SRC management system to support and manage SRC in the acute, chronic and long-term phases.

## 1 Introduction

Gaelic games are hugely popular games in Ireland which are played across all age demographics and are very popular spectator sports which enjoy a growing global audience (1). The history of these sports, such as hurling can be traced back to ancient times, with some estimates suggesting the game is over three thousand years old (1). The predominant forms of field-based Gaelic games are hurling, Gaelic football and camogie, which is the female counterpart to the game of hurling. These games are classed as intermittent invasion games and are recognised as fast paced, skilful and physically demanding (2). The governing body of Gaelic games are the Gaelic Athletic Association (GAA) who coordinate and manage all Gaelic games. One unique facet of the GAA and Gaelic games is that there exists a two-tier grading for adult athletes who participate. These include ‘amateur’, who solely play with community-based clubs and an ‘elite amateur’ grade, who represent their county (region) at intercounty competitions. Typically intercounty games (elite amateur) are longer in duration, they are played at a faster pace and the players are highly physically conditioned athletes (3).

As is common in many field-based invasion games, physical contact is inevitable and will expose players to injury risk such as musculoskeletal injuries and other injuries such as sports related concussion (SRC) (4). SRC is prevalent in a range of sports and is a growing concern in contact sports including Gaelic games (5, 6).

Regardless of whether athletes are playing amateur or elite amateur, they remain exposed to injury risk on a consistent basis, including injuries such as SRC (4, 7, 8). In recent years, there have been multiple efforts from many national governing bodies to increase SRC awareness. However, the current management of SRC in amateur sports remain inconsistent, and there may be potential long-term implications for athletes’ health due to non-disclosure of SRC incidence (9, 10).

As many incidences of SRC either go undiagnosed or unrecognised, it is important to note that many athletes feel pressure to return to play quickly (11). These pressures can manifest due to the competitive nature of sports and the fear of losing their place on the team (12). As has been investigated by previous research, a premature return can exacerbate symptoms and prolong recovery (13). A complete recovery from SRC involves more than a physical healing process; it requires a multi-faceted approach, including managing cognitive, emotional, and social aspects (14).

Access to medical professionals regarding SRC management in amateur settings is often limited (15). In the absence of suitable medical guidance, athletes may not follow recommended protocols, or not disclose their symptoms which may lead to incomplete recovery (16). Although many instances of SRC can resolve in a relatively short period of time, there are circumstances whereby athletes can experience persistent physical symptoms, such as dizziness, fatigue, or headaches (17). These physical symptoms can endure for months, and in some situations, the symptoms can last for years which may have a long-lasting effect on an athlete's quality of life (18).

Due to the complexity of SRC, some athletes may suffer long-term cognitive effects which can affect an athletes memory, attention span, and executive function (19). Some athletes can be exposed to emotional symptoms, which can range from irritability, mood swings to depression (20). These impacts can lead to added frustrations and a diminished sense of purpose, which may further exacerbate emotional and psychological distress these cognitive and emotional concerns can interfere with activities of daily life and academic or occupational performance or to participate fully in social activities (21).

Due to the pervading amateur ethos embedded in Gaelic games, it can lead to different teams (amateur or amateur elite) often having varied approaches to concussion management, these different approaches can lead to inconsistent care. Without standardized protocols, teams may not uniformly enforce or follow recommended SRC return to play (RTP) guidelines. This lack of comprehensive guidelines may contribute to a highly varied approach across different levels of Gaelic games (22).

As all athletes in Gaelic are expected to maintain an amateur/elite amateur status, the athletes themselves exhibit a remarkable loyalty and commitment to their clubs (amateur) and counties (elite amateur). This dedication is exemplified by an unwavering participation in prolonged playing seasons, training sessions, competitive games, which in turn can lead to player burnout (23). This discipline is amplified in elite amateur settings where this ’semi-professional’ approach is evident in their approach to county training, high pressure competitions, and conduct both on and off the field.

As there is a paucity of research in the area of SRC and post concussive syndrome (PCS) within the GAA. The present study seeks to gain insights into the current state of concussion recovery in Gaelic sports from the athletes’ perspective using qualitative research methods. These methods aim to widen the understanding of SRC and PCS in Gaelic games and enhance previously developed quantitative data.

## 2 Methods

### 2.1 Study design

This study employed a grounded theory methodology (24) to investigate the perspectives of current and retired GAA athletes on concussion symptomology in their respective sports. The research focused on their views on personal concussion symptoms and managing long term effects including possible SRC induced retirement. Data were collected through semi-structured interviews (25).

All participants had experience playing Gaelic games at either the amateur or elite amateur level. Most athletes remain involved in a playing capacity within Gaelic games at amateur or elite amateur level. The interview questions were designed to elicit responses about their experiences regarding SRC symptoms, discuss long term effects and understand their opinions on the expected commitment within Gaelic games. Pilot interviews were conducted within the wider research team to check for interview question clarity and to ascertain an average of the overall duration of typical interviews.

### 2.2 Ethics & procedure

Ethical approval was granted for this research via the Research Sub-Committee of Academic Council of Atlantic Technological University (ATU). Opportunities were offered to the participants to engage in a pre-interview meeting. These meetings aimed to clarify the purposes of the study and how their information would be secured and managed in a confidential manner. All data were collected during scheduled online interviews (via Microsoft Teams). These semi structured interviews ranged from approximately twenty minutes to sixty-one minutes in duration. In advance of the interviews, each participant was provided with a comprehensive participant information document. Once the participant had satisfied all pre-interview issues, the interviewer sought written consent prior to their allocated interview. In addition, as a final confirmation prior to the interview, the interviewer requested verbal permission. Once the participants had no further questions, the interviews continued using a set of standardized broad and specific questions which were used for all interviews in this research. At the end of the interview, it was stated that all information provided would be treated with the utmost confidentiality and completed anonymised for research purposes.

### 2.3 Participant characteristics

The participants in this research (*n* = 22) have all participated in one or more Gaelic sports at varied levels. Most participants (*n* = 18) remain active at either amateur or elite amateur, and the remaining (*n* = 4) are retired from Gaelic sports. The study used a snowball sampling methodology as the members of the research team were based in Ireland. The following counties were represented; Tyrone (*n* = 3), Clare (*n* = 7), Tipperary (*n* = 1), Cork (*n* = 1), Limerick (*n* = 1), Galway (*n* = 4), Wexford (*n* = 2) and Mayo (*n* = 3). From the group, 82% (*n* = 18) have or had represented their respective counties at elite amateur senior level in their respective Gaelic sport.

The cohort was comprised predominantly of males (64%) and with eight female participants (36%). The cohort had an average age of 31 (SD ± 5) years old and 28 (SD ± 5) years old for male and female participants respectively. All participants in the group had a history of at least one medically diagnosed concussion. Although, many participants were uncertain about the total number of concussions they had experienced, either diagnosed or undiagnosed.

### 2.4 Sampling and eligibility criteria

This study used a snowball sampling method where the first participants who agreed to be interviewed provided referrals for subsequent interviews (26). The interview process continued until the point of data saturation occurred. At frequent times during the process, participants were reminded that they were under no commitment to suggest additional participants to the research team.

All participants provided referrals to further athletes who were communicated with and asked to take part in an interview. The structure of the interviews enabled the research team to recognise mutual themes from the interview transcripts. In doing so, the athletes explained their personal experiences of SRC symptoms.

### 2.5 Data collection

The purpose of the study was to understand the current environment of concussion recovery in Gaelic sports from the perspective of the athletes. The study intended to offer the ‘Gaelic sports athlete perspective’ which may provide suggestions for Gaelic games about the long-term effects of mismanaged SRC in amateur and elite amateur settings.

### 2.6 Data analysis

#### 2.6.1 Transcription

Recordings were generated along with transcripts of the interviews using MS Teams (Microsoft, USA). These original transcripts were verified, checked, and transcribed by the lead researcher (ED). Following this initial assessment, the transcripts were analysed for correctness against the MS Teams video recordings. In turn, these were then rechecked using an audio recording to identify audio errors (e.g., pronunciation of words in regional accents) and eliminate any subtle errors in the transcripts.

#### 2.6.2 Decontextualization

The process of decontextualization involved listening to an audio recording while reading each transcript (27). This process enabled the research team to confirm the sequence of interview questions and determine if the questions and answers compared with the audio files of the interviews. This process was performed on 4–5 separate occasions with each transcript and recording by the research team. In addition to this process, the research team utilised research interview notes to support the development of emerging patterns and themes.

#### 2.6.3 Recontextualization and categorisation

The lead researcher ascertained individual themes and subsequently, these themes were divided into categories, subcategories from the interview transcripts (28). Table 1 displays the reported athlete SRC symptomology in acute, chronic and in the long-term phases. In addition, Table 1 displays athletes’ issues such as forced retirement from their sport, or experienced long term SRC side effects.

**Table 1:** Sample results of thematic analysis of interviews with Gaelic games athletes (*n* = 22).

Themes	Categories	Subcategories	Sample quote
Male and female athletes experience a range of acute and chronic symptoms post-SRC	Symptomology, unknown side effects and long-term effects	Acute SRC symptoms; symptomology in male and female athletes	“I couldn't even walk because it would give me a headache, so I was literally went from exercising every single day to lying on a bed and trying to keep my eyes open”. (P10)
		Chronic symptomology	“Concentrating like this now “one to one” (the interview), its often hard to hold a conversation, I feel lightheaded or it's just not like fully being concentrated” (P15)
		Long term effects	“I think it had a kind of a psychological impact on me, in that sense that I've was really, like, shook and panicked, first of all, frustration, and then any sort of threat of getting hit that again sent like shocks and chills down my body”. (P16)
Gaelic sports athletes are expected to demonstrate constant allegiance and commitment to the GAA	Commitment to the sport, injury risk and dejection	Commitment to Gaelic games and the accepted injury risk.	“Obviously players at the top level, are more and more professional and there's probably a lot more going on as regards guys monitoring their health and things like that off the field and on the field”. (P7) “Even when I got my first one or two it was similar enough like it was you were at the next training session, and you continued on (P9)
		Perceived dejection by peers or management once long-term injury manifested.	“I suppose the player, the personal point of view, theyve given everything that they have done to the sport. What have they gotten from it? Like I find once you're not playing, you're not wanted, or you're wanted to give back”. (P11)

### 2.7 Coding

This research used a semantic interpretation for all the interview transcripts (28). This iterative process was performed by the lead researcher (E.D.) and further assessed by the second member of the research team (L.R.). The primary codes encompassed a wide variety of SRC symptomology from the athlete's perspective. Themes were classified by the lead researcher (E.D.), and these classifications were discussed in greater detail with the second member of the team (L.R.). Subsequently, categories and subcategories were created to represent the connected interactions.

## 3 Results

In this study, two major themes were identified; (1) SRC symptom recovery experiences in male and female athletes and (2) Expected allegiance and commitment from Gaelic sports athletes (Table 1).

### 3.1 Theme 1—male and female athletes experience a range of acute and chronic symptoms post-SRC

#### 3.1.1 Acute SRC symptoms; symptomology in male and female athletes

Possessing little or no SRC knowledge prior to experiencing an incident was a prevailing view held by the majority of participants. This may have been on account that they had little personal exposure to SRC, or simply they had never been informed that SRC was a prevalent injury in their sport, “*I think a lot of people think you have to lose consciousness to be concussed and I think it's trying to dispel that myth*” (P1). This widespread misunderstanding of SRC, and a vacuum of information about SRC as an injury became abundantly clear based on observations from the cohort of athletes regarding SRC management, “*I can get past this stuff (concussion symptoms) and it wasn't a new occurrence to me*” (P6), “*I remember one of the lads says, you literally just laid down in the car, and you're just stared at the road the whole way home*” (P8) and “*By this stage there was no feeling (of being) sick or vomiting, and my girlfriend didn't sleep all night, she was just afraid*” (P9). These examples of self-managing an SRC, to acute memory loss or not knowing how to manage acute SRC symptoms were common across all participants in the research cohort.

Mismanaged acute symptoms may have placed the athlete in a position of immediate danger to themselves or others,

*“I met the first car and pulled in and I was giving out saying ‘what are you at’ on the other side of the road. I thought she was on the other side of the road, and then went around the corner, met another one (car), and then I knew I was wrong then, because I hit the curb”* (P15)

and “*I'd still find little triggers….definitely (there) was a big issue, even driving in the dark or especially driving in the dark when it's raining hard* (while symptomatic)” (P18).

Many participants possessed anecdotal knowledge of what constitutes an SRC, whereby they reported “not feeling themselves”, being “in a fog” or feeling fatigued. There was a broad range of acute symptomology experienced by male and female athletes. For example, one male athlete reported a “*dizziness and the disorientation, tiredness….fairly substantial for a couple of days, like it was, on screen, yeah, screens and that, like in my eyes, sensitivity to light*” (P9) and a female athlete stated regarding acute symptoms

*“Headaches throughout, tiredness throughout, but some days I'd wake up and I'd just be really like, feel not, well, feel like I could get sick. And then I could be fine the next day, then I could be bloated. There was no kind of sequence of events to it (ongoing symptoms)”* (P10).

A common experience for many athletes in the acute phase were feelings of nausea, one male athlete commented “*That pressure in my head would have been the major one…; I thought if I stood up quickly, I would get sick everywhere if I moved my head quickly, I'll get sick everywhere*” (P5). Similarly, a female athlete stated that

“*My head was spinning, got back to his (boyfriend) house, I was like puking. Very nauseous. This was a vestibular concussion, so I was like, like, my eyes were very affected. So, light. I literally just wanted to sleep in a dark room*” (P17).

In addition to these examples there were other common SRC symptoms in male and female athletes such as emotional disturbances, “*I was walking around without actually falling apart from random bursts of like tearfulness and just like out of nowhere I would just like I would start to cry, or I would be very teary*” (P5).On occasions many symptoms displayed in tandem, “*They (migraines) got a little bit longer, so after the first one, the first one again like felt fairly standard it was nausea for a few days after and a little bit of dizziness*” (P18). Interestingly, one acute symptom which was solely evident in a number of the female participants was that “*spots came out of my scalp, and I felt like there was stuff moving in my hair, like I was scratching my hair for, I'd say the 3–4 weeks afterwards*” (P14), which in turn evolved into a series of chronic symptoms.

#### 3.1.2 Chronic symptomology; post-concussion syndrome

Due to the absence of appropriate information regarding the side effects or long-term effects of SRC, many of the athletes felt that their personal recovery was their responsibility alone, “*I monitored it myself and I kind of had an idea of what to look out for I suppose*” (P10) and “*I remember going and looking for information (post-concussion), I think at the time it was the NHS (UK) website I was on looking at symptoms and stuff”* (P13). Even where the information was coming from a medical source, the information may not have been reflective of current SRC practices “*everybody would have been telling me; all the doctors I would have known….you just need to rest and give yourself time*” (P5). These situations clearly exacerbated underlying chronic symptomology such as effects on the athletes’ mental health

*“The mood was a big one, that was affected. It decreased (mood) a lot the weeks following it, like between, you know, not having your outlet of hurling and like, you know, not knowing what's gonna happen and then actually like your mood just in general would be up and down for no reason”* (P2).

It is arguable that these serious side effects had detrimental on athletes’ mental state “*I didn't realise (mental state) had anything to do with concussion, I was feeling really kind of low and sensitive”* (P18). These effects on mood likely reflected a deeper concern based on the inability to take part in group training or competitive games “(*I was) doing minimal physical activity like for the first two weeks, all I was allowed to do was exert my eyes or go for a walk, so, like, it really affects your mood”* (P17) and “*I couldn't even walk because it would give me a headache, so I was literally went from exercising every single day to lying on a bed and trying to keep my eyes open*” (P10)

This added sensitivity amplified previously unnoticed events which happened on a daily basis

*“Even the smallest thing would annoy you. Someone clicking their pen or even just any noise people talking, just get at you, it was like they were attacking you, rather than it just being something that you're so used to when you're in normal form or when things are fine”* (P22).

Another aspect worth considering are the unpredictable bouts of chronic SRC effects, most notably these occurred during random acts of daily living

*“When I was out with the dog (walking) and my legs just stopped moving, that was a scary point. I was like ‘actually this this is serious’, and I started saying it was a brain injury”/I really had to tell my brain to tell my body to do simple things like move and talk, (getting) headaches when I've got stressed”* (P14).

What was certain is that many athletes experienced the chronic effects of post-concussion syndrome or persistent concussion symptoms. It was obvious during the interviews that athletes were able to delineate between short term and long-term symptoms, particularly after experiencing a series of SRCs, or suspected SRC “*Definitely after the third one, the symptoms are still ongoing for months afterwards*”. (P18).

#### 3.1.3 Long term effects on personal life, education, and occupation

During this current research, it was remarkable to discover how many of the cohort continued to experience long term effects of their SRC(s). In many instances, both male and female athletes are managing lifelong tangible effects of injuries inflicted by Gaelic games,

*“I got knocked in that eye, when I come into really close range of something, it (involuntary eye movement) swings out to the right and it still does that now….its three years on now and my eye never came back to normal”* (P16).

Another athlete reported direct effects after multiple untreated SRCs that “*If I spin around two or three times (playing with his children), I'm nearly holding on to something, my orientation is not good with bouncing or spinning and like it (vestibular function) was always perfect*.” (P9). Even without irregular movements, some participants are experiencing ongoing, long-term issues, such as “*That's one that sort I just have to live with (light sensitivity), you know, and believe it or not, even in the not sunniest days, I'll probably still wear sunglasses*” (P1)

These long-term experiences were not limited to oculomotor or vestibular function. Some athletes have noticed a progressive deterioration in their capacity to tolerate minor irritations on a regular basis “*(I) would have been probably a lot more irritable and for sure, I probably have struggled with that over the last few years”* (P4). For some participants, multiple SRCs or sub concussive impacts have had reported negative effects on their capacity to memorise or retain information “*The other thing I feel with the long-term symptoms….but I do feel my short-term memory is absolutely terrible since (the concussions) and obviously you know, I can't prove that there are any links to concussion”* (P18), further supported by “*I'm more tired, maybe mid conversation at times, I'm scratching my head going….I've just noticed. I'm not. I'm still not right….until this day I still, with numbers, sometimes I'll struggle to remember more than three digits, you know*” (P1). Memory may be treated as an invisible attribute and may go unnoticed, long term external changes can occur which may be more overt to the observer after experiencing multiple SRCs over a reportedly short duration “*I'd ask a question once or maybe twice where before, I never would have, and now they (family) make out that there is a slight slurring of my speech as well*” (P12) and “*I had a shake in my right-hand arm for about twenty minutes and it spooked quite a few of the boys. You know, it actually looked like I had Parkinson's in my right-hand arm*” (P1).

Some participants discussed alterations in the work life to accommodate ongoing symptoms, where they were forced to cancel paid work or seek extra time off work due to a recurrence of symptoms “*I would have definitely had to take time off work because looking at the screen and reading stuff like reading small print”* (P14) and “*I was going to be working that Saturday night and I actually do remember talking to my boss and I couldn't go into work*” (P17), even while at their occupation, they were still feeling unfit to work or maintain full attention during their working days “*trying to like in the middle of meetings, when they were thirty minutes or more, (I was) losing concentration, and being very fatigued during them (work meetings)*” (P2).

Because many of the athletes who were interviewed expressed concerns over their long-term health, they had an uncertain outlook. The vast majority of the comments about these health effects in the long run were influenced by what they had read or heard regarding multiple SRCs, “*I think that (multiple concussions) tallies with my experience, and it also tallies with the research there for sure, so yeah, I'm pretty certain that is the that is the case (long term consequences*)” (P4). There were anxieties about what may happen in the future, and how this could be damaging to them “*if you were to, you know, constantly or consistently have knocks to the head and you have these symptoms that they can persist and they can be difficult to reverse or irreversible as the case may be*” (P13). Due to a dearth of knowledge from their perspective, many athletes were uncertain about what repeat SRCs meant for their personal situation, “*I'd imagine there would be, you know serious long-term effects if you kept playing. If you didn't recover properly and then went playing and got a belt again? I said that's the real risk.”* (P20), and “*I just think that more you have, especially if they're close in succession, I think, yeah, I definitely think so (long term effects)”* (P9)

### 3.2 Theme 2—Gaelic sports athletes are expected to demonstrate constant allegiance and commitment to the GAA

#### 3.2.1 Commitment to Gaelic games and the accepted injury risk

Because of how Gaelic games are embedded into the cultural landscape of Ireland, participants begin playing these games at a young age, circa four to five years old. In conjunction with these early beginnings, lies a central connection to the athletes’ local club. These factors instil a deeply held sense of commitment to the sport and to the area from which the athlete was raised., “*(I) played for my local club, since I was six or whatever, you know, and played the whole way up to senior level”* (P1) and “*I think the memories and the people you meet over the years are probably the highest (accolade) actually that you could ever, ever achieve on it, you know, so very grateful*” (P3).

These expected levels of commitment to club and county teams was viewed as a “privilege” and athletes saw themselves as ‘lucky and ‘fortunate’. Due to these perceived circumstances, some felt that they may have limited opportunities to reach the highest levels of their sport “*When I was my younger self, you're probably putting a lot more pressure on yourself because you feel like, this might be the only chance you might get”* (P21). These were commonly the occasions where they may expose themselves to further injury risk, “*I knew in my heart and my soul that I wouldn't be able to play the game. But at the same time, I was just very hopeful that I my symptoms would improve by then*” (P17). Athletes were willing to play through SRC, even though they knew the risks involved “*I was like ‘oh don't really feel too great’, I like took a second, to kind of get my bearings, and a little bit of ringing in my ears because they had hit me in the side of the head”* (P13) and “*But like in our sport, you'd stick your head where you wouldn't stick your hurley, and you'd have a pain in your head”* (P11).

A very common opinion was that the athletes were willing to continue playing through SRC, this was particularly evident in the elite amateur grade of Gaelic games, “*you're in the middle of that match, especially in front of 50,000 people, and you're playing probably the best game, you know in your head….you don't want to leave*” (P6), supported by “*something that just keeps me going, I suppose, once you've played in Croke Park* (national GAA stadium)” (P15). In many cases, because the competition was fiercely intense, athletes did not want to be seen to stop training, even while they were evidently symptomatic, “*Sometimes the way my symptoms would have come on was when you're doing your absolute highest intensity activity…. that's the time you don't want to be seen to drop out of something, you don't want to be seen to stop”* (P18). As mentioned previously, all participants had experienced at least one SRC, however the norm was that the majority had endured multiple SRCs. In tandem they had witnessed fellow athletes suffer SRC while they were playing,

*“You'd nearly be telling the player to go off, like, just go off and, as elite athletes in a sense, like no one wants to go off in case you lose, I don't think people understand how serious it is to play on either”* (P10).

#### 3.2.2 Perceived dejection by peers once long-term injury manifested

In amateur and elite amateur sport, everyone's career ends for various reasons such as chronological age, loss of form, changes in management structures and through long term injury. A permeating opinion throughout this research has been a sports career ending with something intangible such as an SRC. Many athletes can accept a catastrophic musculoskeletal injury, for example cruciate ligament damage or Achilles tendon rupture. Chronic symptoms related to SRC are perceived as being more difficult to rationalise as SRC is viewed as an “invisible injury”.

In accordance with the expected level of commitment to the sports, athletes were expected to play through injury, otherwise, they risk being perceived as “soft” or “weak”, “*In the past it might have been nearly seen as if you're being a bit soft, there was nearly a narrative there that it (SRC) isn't that serious even though it actually is*”. (P22) and “*It was literally get on with it unless you were to fall…… and just couldn't physically get up like, you know, or it was probably seen as being weak*” (P8). There was a sense of palpable pressure from a number of the participants. The sources of the pressure were self-imposed, apparent expectations from their peers or management, and an intensified pressure of not being able to perform for their clubs or county.

These pressures escalated when athletes were compelled to retire from Gaelic sports due to multiple incidences of SRC. These individuals found themselves in a state of confusion and frustration, “*that sort of nine-month period was where peaked in terms of the amount of events or occurrences of concussions and that left (me), very cautious and hesitant player”* (P4). When athletes, willingly or unwillingly decided to retire, they felt that their lifelong commitment to Gaelic sports was quickly forgotten about and resulted in athletes to become irate and frustrated,

*“You're very much, I think, forgotten about. If you can't be on the field or coaching beside them…they've given everything that they have to the sport. What have they gotten from it? Like I find once you're not playing, you're not wanted”* (P11).

A percentage of these frustrations were based on an inability to explain SRC in terms of the real effects it was having on their lives, “*I do think in general it is viewed differently. It's one of those invisible injuries ….that they're (symptoms) hard to kind of explain*” (P18). This difficulty in explaining long term symptoms, when on first appearances to the observer, the athlete was not visibly injured, for example they were not using crutches, or need a joint brace, resulted in added stresses for the athletes.

Even though Gaelic sports promote an image of support and collegiality, the lived experience for some was not reflective of these principles, “*The culture of the GAA, even though it's this whole grounded community volunteer basis….I suppose it's an important point to add(that) its results driven, so I think that alone tells the story regards of what injury you may have*” (P11). The inference from this statement meant that if the injury was visible, then peers and management would be more likely to accept a full recovery timescale. This was generally not the case for athletes who were managing long term SRC symptomology, “*So therefore, maybe the friendship was false because the only reason we were friends was because of football, you know*” (P1) and further supported by “*They (the hurling club) don't give a fuck about how you can't remember things in your sixties and seventies. If you have a couple of medals, you're great like”* (P12).

Some athletes discussed attempts to “make a comeback” after recovering from a SRC, or while experiencing long term issues associated with SRC. One athlete had extended bouts of symptomology, however, believed “*if I did get another bad concussion, that would be it. I'd stop playing contact sport if I got another one, I'd say*”(P10). Another athlete attempting to return to play after enduring long term symptoms stated they were “*Still not feeling right, headaches, and when I run I do it. (I’m) getting constant headaches, stuff like that”.* (P19). In the midst of these examples, some athletes were accepting that their Gaelic games career had ceased indefinitely “*I'm kind of enjoying the break (from inter county camogie) because it's so intense and when you're on it, it kind of takes up your whole life….and I kind of realize that there's more to life”* (P16)

## 4 Discussion

Comprehensive SRC management represents a challenge in contact sports, particularly sports where an amateur ethos is promoted and instilled such as Gaelic games (29). While many of the immediate effects of SRC are relatively well-documented, the unseen and often under-recognized impacts on athletes’ health, both physical and psychological, warrant further exploration (30). The purpose of this study sought to examine these unseen impacts of SRC, and the subsequent issues related to the recovery processes with an emphasis on the lesser understood long-term consequences of mismanaged SRC.

It is well established that failure to diagnose or mismanage any injury will be detrimental to an athlete's sporting career (31, 32). From this perspective, the mismanagement or misdiagnosis of SRC can have severe and long-lasting consequences for athletes (33). It is well documented that where there are inadequate management practices of an SRC, these poor practices can result in prolonged recovery periods (34). Athletes may experience persistent symptoms, such as PCS, which can last for months or years. PCS includes chronic headaches, cognitive difficulties, emotional instability, and sleep disturbances (35). These chronic PCS symptoms can significantly impact an athlete's quality of life, their academic performance, or their ability to work (36). Further serious complications may arise when athletes (amateur, elite amateur and professional) may develop significant mental health issues, including anxiety, depression, and post-traumatic stress disorder (37, 38). The psychological impact of long-term symptoms and the associated social isolation from sports and peers can exacerbate these conditions (39).

For amateur athletes, there may be a risk of developing long-term cognitive and psychological issues via the cumulative effects of undiagnosed or mismanaged SRC (40). This underscores the importance of effective concussion management protocols and an organisational culture which prioritizes athlete health and safety over competitive success (41).

The culture embedded in Gaelic games plays a crucial role in shaping attitudes towards injury risk and concussion management. This cultural backdrop can create an environment where injuries are downplayed or normalized, and athletes might feel pressured to continue playing despite being concussed (42). Historical perspectives on sports injuries within contact sport often elevate athletes who play while injured, viewing it as a demonstration of an athlete's loyalty and resilience (43). This can lead to underreporting of SRC, or misdiagnosis of SRC and subsequently inadequate recovery, as players fear losing their place on the team or disappointing their peers and coaches (44). Efforts to change this culture need to be implemented with an emphasis on the serious long-term effects of SRC (9). Athletes who endure long-term unseen symptoms, often face psychological and social challenges associated with SRC. In addition, it's imperative that there is an attitude shift by reducing the stigma associated with reporting and treating SRC (45).

The perception of dejection or rejection by their fellow peers and management can exacerbate the emotional and psychological toll of the SRC (46). This is particularly acute in Gaelic games, where community and team identity (club and county) are intertwined (47). In many cases, injured athletes may feel isolated from their teammates and the sport they have oftentimes dedicated their lives towards. The harsh loss of their athletic identity, coupled with the fear of being seen as weak or dispensable, can lead to feelings of depression and anxiety (48, 49). The perceived lack of support from coaches and peers can further compound these issues, making recovery from SRC even more challenging (50). Organisations should foster an accepting and supportive environment for athletes who are experiencing acute, chronic and the long-term effects of SRC.

## 5 Conclusion and limitations

SRCs are a considerable injury concern for both male and female amateur and elite amateur Gaelic games athletes. Although the cohort of athletes in this study provided a depth of previously unseen insights into the lack of structured SRC management in Gaelic games. A significant limitation, which was beyond the remit of this study, were the notable shortages of literature acknowledging the complex topic of SRC in Gaelic games. In general, most academic literature which discussed injury management and reduction in Gaelic games, rarely mentions SRC let alone the myriads of ongoing side effects experienced by athletes at amateur or elite amateur levels. This large gap in the literature requires immediate and comprehensive research to elucidate these unseen and unmentioned phenomena.

Based on this current study, SRC has the potential to create both acute, and long-term health consequences. Acute SRC management, and assistance toward the management of chronic symptomology require a more meaningful and comprehensive approach by the GAA. This may begin by attempting to adjust long held, deeply entrenched cultural attitudes towards SRC. This process can be instigated by creating a supportive environment for athletes at all levels within the GAA to disclose SRC. There is an immediate need to prioritise well supported SRC education for athletes, coaches, referees, and medical staff to ensure proper management, and psychological support. By confronting and accepting these issues, then the Gaelic games community can better protect its athletes and promote a healthier culture.

## Data Availability

The raw data supporting the conclusions of this article will be made available by the authors, without undue reservation.
